# Quercetin as an innovative therapeutic tool for cancer chemoprevention: Molecular mechanisms and implications in human health

**DOI:** 10.1002/cam4.1411

**Published:** 2019-09-30

**Authors:** Rafiq A. Rather, Madhulika Bhagat

**Affiliations:** ^1^ School of Biotechnology University of Jammu Jammu and Kashmir India

**Keywords:** Apoptosis, cancer, cell cycle, chemoprevention, quercetin, signaling networks

## Abstract

Cancer is a life‐threatening disease afflicting human health worldwide. Recent advances in drug discovery infrastructure and molecular approaches have helped a lot in identifying the novel drug targets for therapeutic intervention. Nevertheless, the morbidity and mortality rates because of this disease keep on rising at an alarming rate. Recently, the use of natural and synthetic molecules as innovative therapeutic tools for cancer prevention has lead to the development of cancer chemoprevention. Cancer chemoprevention is a prophylactic strategy that involves the chronic administration of one or more natural or synthetic agents to block, to inhibit, or to suppress the process of cancer development before it becomes an invasive disease. Quercetin, a dietary bioflavonoid, can specifically retard the growth of cancer cells and behaves as a potent cancer chemopreventive agent. Quercetin has multiple intracellular targets in a cancer cell. Therefore, many mechanisms have been postulated to explain its chemopreventive action. The chemopreventive effects elicited by this natural molecule in different model systems are believed to include antioxidant/pro‐oxidant action, regulation of redox homeostasis, apoptosis, cell cycle arrest, anti‐inflammatory action, modulation of drug metabolizing enzymes, alterations in gene expression patterns, inhibition of Ras gene expression, and modulation of signal transduction pathways. However, cell signaling networks have recently garnered attention as common molecular target for various chemopreventive effects of quercetin. In this review, we made an attempt to critically summarize the emerging knowledge on the role of quercetin in cancer chemoprevention and the underlying molecular mechanisms implicated in its chemopreventive and therapeutic effects.

## Graphical abstract

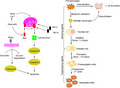

## Introduction

Despite decades of research, median survival times of cancer patients have not improved significantly. Notably, in 2017, 1688780 new cancer cases have been projected to occur in the United States 1. Various dietary, environmental, genetic, and epigenetic factors contribute to cancer development in high‐risk individuals 2. Diet represents approximately 30 to 35% of risk factors that can contribute to the development and pathogenesis of cancer 3. Therefore, cancer development can be retarded to a large extent through appropriate dietary modifications and limiting exposure to dietary and environmental carcinogens 4. To date, no “miracle molecule” exists that can cure any form of cancer. Nevertheless, phytochemicals, non‐nutrient components in the plant‐derived foods, have garnered attention for potential cancer chemopreventive properties and safety profiles 5. However, understanding the mechanisms underlying the chemopreventive effects of phytochemicals is mandatory before they can be recommended for inclusion in human diet or tested in animal models/human intervention trials 6.

Cancer chemoprevention is meant to inhibit or retard the process of cancer development 7. The selection of an appropriate chemopreventive agent was initially based on epidemiological experiments, where it has been reported that consumption of particular dietary component (e.g., quercetin) can reduce the incidence or mortality of a specific cancer (e.g., lung adenocarcinoma) 8, 9. Over the recent years, the strategies of developing a novel chemopreventive agent have changed markedly. Now, extensive preclinical mechanistic studies of a lead molecule are conducted before clinical trials are recommended and specific biomarkers for early prediction of drug efficacy are given ample attention 10. Chemopreventive agents constitute a structurally highly diverse group of cancer preventive agents, making it difficult to elucidate their structure–activity relationships and underlying mechanisms of action. Therefore, it is advised to examine their effect on cancer‐associated signaling pathways using appropriate experimental models 11.

Single agent chemoprevention which involves the administration of single agent is usually less efficacious than combination chemoprevention by multiple agents 12. It is believed that combination chemoprevention might provide “additive synergism” against cancer development with minimal or no adverse effects 13, 14. The chemopreventive effects of quercetin have been studied both in vitro and in vivo 15. In this review, we provide mechanistic basis of the role of quercetin in cancer chemoprevention and how quercetin influences the cell fate in different experimental models.

## Importance of Quercetin in Foods

**Figure 1:**
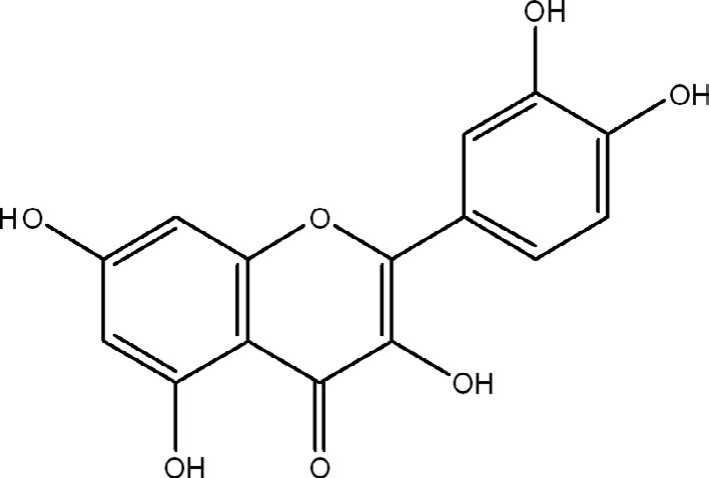
Chemical structure of quercetin.

Quercetin (Fig. 1) is pharmacologically active and safe bioflavonoid widely distributed in onions, apples, broccoli, tomatoes, and citrus fruits 16, 17. Quercetin possesses a series of therapeutic activities such as antioxidant, anti‐inflammatory, antimicrobial, and anticancer activities 18. Quercetin naturally occurs either as glycoside (with attached sugars) or as aglycone (without attached sugars) both of which are biologically active 19. However, poor aqueous solubility and low bioavailability limit its clinical use 20. Quercetin bioavailability is greatly enhanced when it is ingested as an integral dietary component in the form of fruits and vegetables.

## Mechanism of Cancer Chemoprevention: An Overview

**Figure 2:**
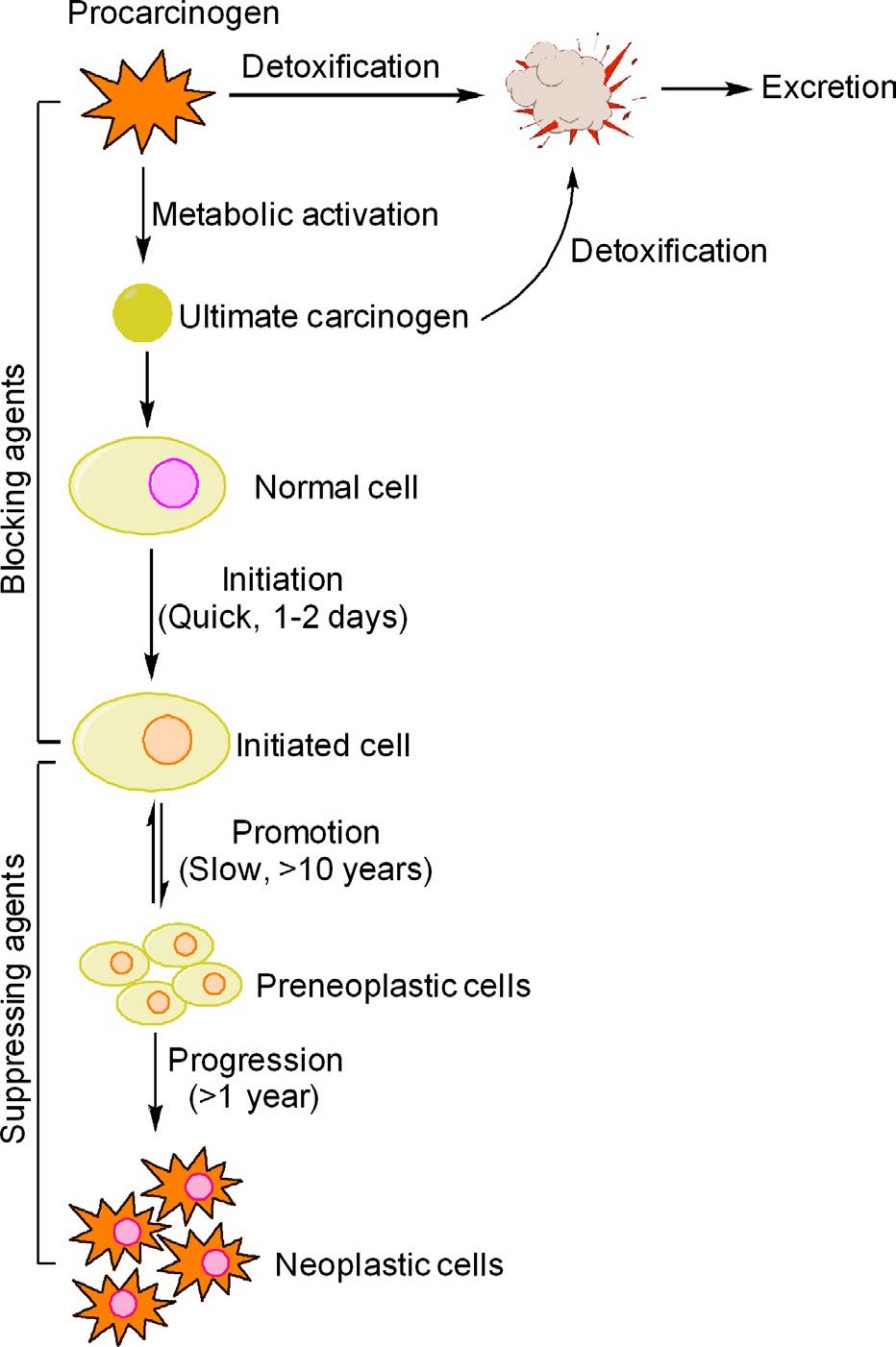
Stages of cancer development and chemopreventive targets. Cancer development is a multistep process that initiates when a normal cell begins to behave abnormally and transforms into an initiated cell. Tumor initiation is followed by tumor promotion where initiated cells acquire multiple genetic and metabolic changes over a considerable fraction of an individual's lifetime, resulting in formation of preneoplastic cells, which finally progress to form neoplastic cells. Each of these stages provides ample opportunities to interfere with cancer development. Certain chemopreventive agents interfere with metabolic activation of procarcinogens into ultimate reactive forms and block their interaction with DNA,RNA, and proteins. These agents are known as blocking agents because they block tumor initiation. Some blocking agents stimulate detoxification of carcinogens and facilitate their removal from the body. Another group of chemopreventive agents are capable of suppressing promotion and progression stages of cancer development and are therefore known as suppressing agents.

**Figure 3:**
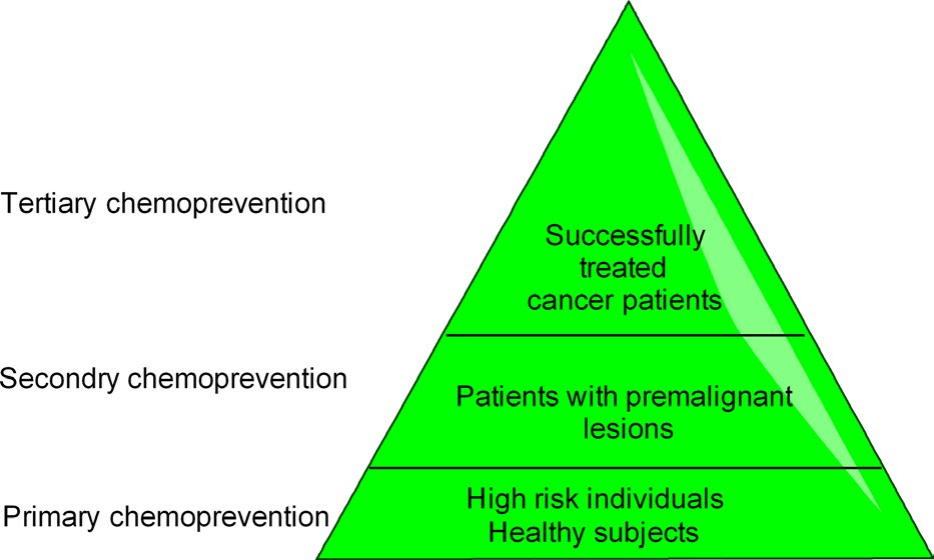
Levels of cancer chemoprevention in clinical settings.

Chemoprevention is based on the idea that cancer is a progressive disease and various signaling pathways and molecular events associated with cancer development can be targeted for therapeutic intervention 21. Cancer development has been divided into three distinct stages—initiation, promotion, and progression (malignant conversion) 22, 23 (Fig. 2). Tumor initiation is quick and irreversible process, whereas tumor promotion, the second stage, is relatively lengthy and reversible process in which preneoplastic cells divide continuously and actively. The third stage of cancer development progression involves active proliferation of neoplastic cells (Fig. 2). Accordingly, chemopreventive agents have been divided into two categories—blocking agents, which inhibit tumor initiation and suppressing agents, which suppress the neoplastic transformation of initiated cells in advanced stages (promotion and progression) of cancer development 24 (Fig. 2). Some chemopreventive agents exhibit overlapping functions of both blocking and suppressing agents. At the molecular level, chemopreventive agents target different stages/signaling events in cancer development (Table 1). In clinics, chemoprevention can be executed at three levels—primary, secondary, and tertiary 25 (Fig. 3). Primary chemoprevention involves administration of agents to healthy subjects or high‐risk individuals; secondary chemoprevention focuses on administration of agents to patients with premalignant lesions, whereas tertiary chemoprevention involves administration of agents to successfully treated patients who are at risk of tumor recurrence 26, 27(Fig. 3). The ultimate aim of all forms of chemoprevention is reduce the burden of cancer on healthcare system.

**Table 1:** Proposed mechanism of cancer chemoprevention

Proposed mechanism of cancer chemoprevention	
Mechanisms of tumor‐blocking agents	Mechanisms of tumor‐suppressing agents
Scavenging of reactive oxygen species (ROS)	Alteration in gene expression
Modulation of antioxidant defense system	Inhibition of cell proliferation/clonal expansion
Induction of phase II drug‐metabolising enzymes	Induction of apoptosis
Inhibition of phase I drug‐metabolising enzymes	Induction of terminal differentiation/senescence
Induction of DNA repair	Modulation of signal transduction pathways
Blockade of carcinogen uptake	

## Chemopreventive Mechanisms of Quercetin

Quercetin is potent chemopreventive molecule because of its cardinal role in influencing the hallmarks of cancer as well as due to its influence on tumor‐associated signaling pathways 28.

### Quercetin as an inhibitor of cancer‐associated ROS signaling

Oxidative stress imposed by excessive reactive oxygen species (ROS) plays a vital role in cancer development 29. As a “double‐edged sword,” ROS may have beneficial or detrimental outcomes in cancer therapy 30. At physiological low levels, ROS act as “redox messenger” to stimulate cell growth processes 31. However, under oxidative stress, excess ROS can damage DNA and initiate cancer development. Quercetin inhibits diethylnitrosamine (DEN)‐induced ROS‐mediated hepatocarcinogenesis through upregulation of enzymatic (e.g., superoxide dismutase, glutathione peroxidase, and catalase) and nonenzymatic (e.g., GSH and total glutathione) antioxidant defense system 32. Therefore, as an antioxidant, quercetin can scavenge ROS and reduce DNA damage and risk of developing cancer. Because of its total antioxidant capacity (TAC) and high reduction potential, quercetin reduces the formation of ROS and reactive nitrogen species (RNS) in lipopolysaccharides‐stimulated THP‐1 acute monocytic leukemia cells 33.

### Quercetin and regulation of redox changes

Due to defects in intracellular signaling networks, cancer cells usually generate elevated amounts of ROS which leads to a state of oxidative stress and makes these cells vulnerable to pro‐oxidant agents impairing redox homeostasis 34, 35. Quercetin is well known for its antioxidant and cell protective effects. However, quercetin also displays strong pro‐oxidant effects and increases the cellular levels of ROS to cytotoxic levels in B16F10 melanoma cells and many other cancer cells 18, 36. Therefore, quercetin may be used to selectively killing cancer cells and be therapeutically useful.

**Figure 4:**
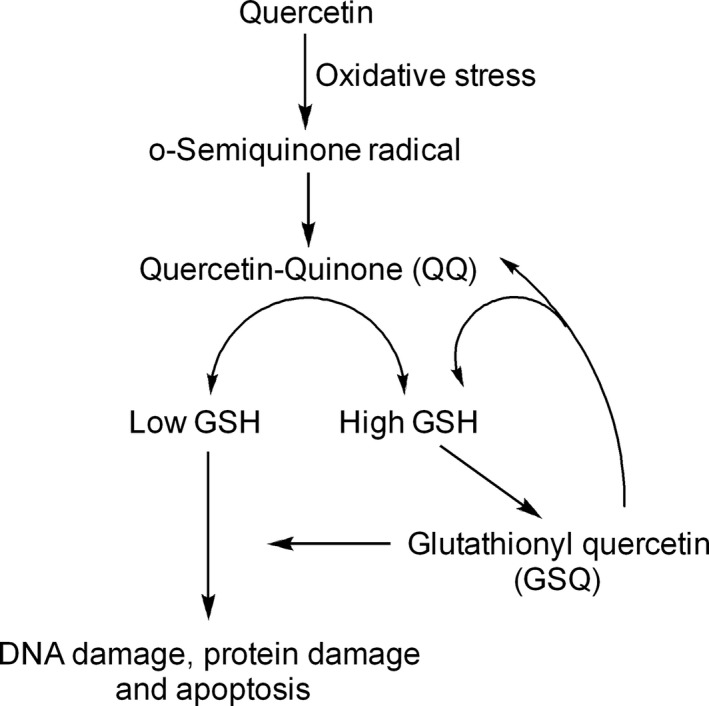
The molecular mechanism of switching between pro‐oxidant and antioxidant effects of quercetin. The switching between pro‐oxidant and antioxidant effects of quercetin is decided by its interaction with reduced glutathione (GSH). During oxidative stress, quercetin reacts with hydrogen peroxide (H2O2) in the presence of enzyme peroxidase to form semoquinone‐radicals which are immediately oxidized to form quercetin–quinone product (QQ). QQ products strong are strong pro‐oxidant and proapoptic moieties that react with protein thiols and DNA to induce apoptosis. Upon reaction with GSH,QQ form glutathionylquercetin (GSQ) adducts such as 8‐GSQ and 6‐GSQ. Interestingly, this reaction is reversible and allows dissociation of GSQ into GSH and QQ. In the presence of high GSH levels, QQ reacts with GSH to form GSQ and QQ again and in this situation QQ does not accumulate enough to induce apoptosis. However, in the presence of low levels of GSH,QQ accumulates and reacts with protein thiols and DNA to induce apoptosis.

The switching between antioxidant and pro‐oxidant behavior of quercetin is often decided by the availability of intracellular reduced glutathione (GSH), a tripeptide redox buffer present in living cells (Fig. 4). During oxidative stress conditions, quercetin reacts with hydrogen peroxide (H_2_O_2_) to form o‐semiquinone radical and quercetin–quinone products (QQ) 37. QQ products are cytotoxic and induce cell death through their interaction with protein thiols and DNA 38. Notably, QQ products react reversibly with GSH to form glutathione–quercetin adducts such as 6‐glutathionylquercetin (6‐GSQ) and 8‐glutathionylquercetin (8‐GSQ) which dissociate persistently back into GSH and QQ 37. In case of high GSH level, QQ reacts preferably with GSH, resulting in the formation of GSQ and in this case, QQ fails to induce cell death; whereas in case of low GSH level, QQ reacts with protein thiols and thereby causes cellular damage and apoptosis. Similarly, extended exposure with high concentration of quercetin causes a substantial decline in GSH levels, impairing the ability of quercetin to scavenge ROS. As a result, pro‐oxidant effect of quercetin overdominates its antioxidant effect, resulting in DNA damage and cell death.

### Quercetin and regulation of cell cycle

**Figure 5:**
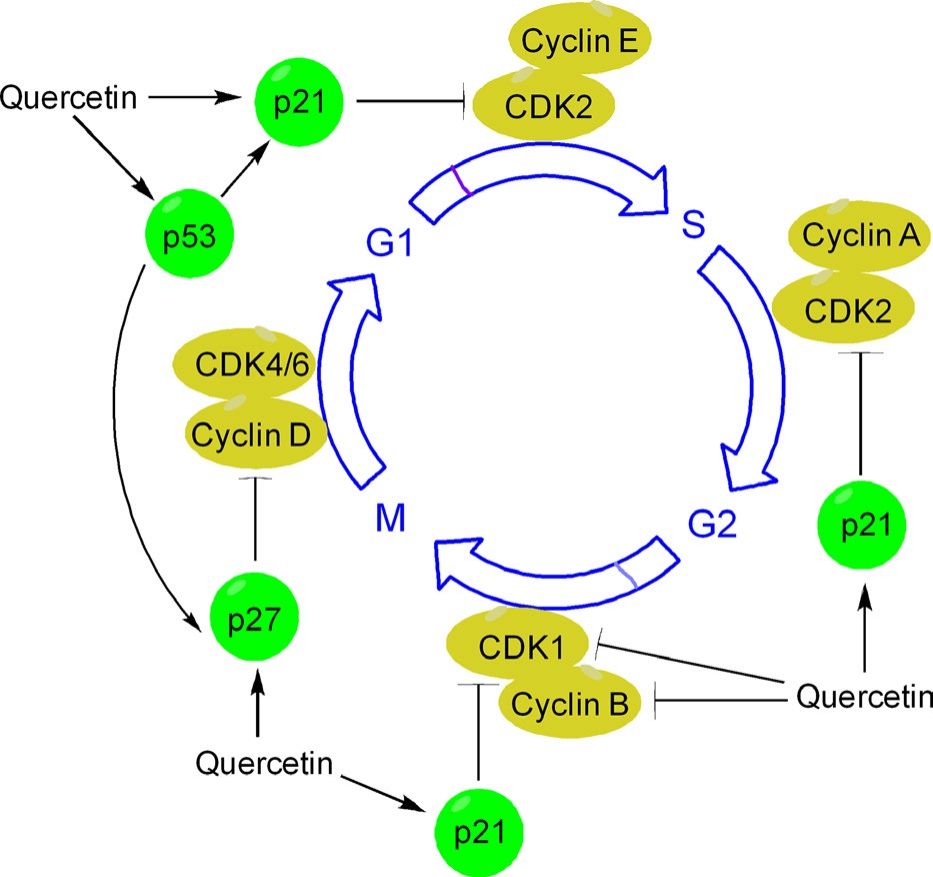
Mechanism of quercetin‐induced cell cycle arrest. Quercetin induces cell cycle arrest in many cancer cells directly by binding to various protein regulators of cell cycle and can affect any of the different phases of cell cycle progression. Quercetin can arrest growth of cancer cells at G1, G1/S or at G2/M phase through its interaction with different regulators. For example, to induce G1 growth arrest, quercetin inhibits cyclin D and upregulates the expression of p21, p27 and p53. p21 is a potent cyclin‐dependent kinase inhibitor and preferentially inhibits the activation of CDK2‐cylcin E complex, leading to subsequent inhibition of CDK‐dependant phosphorylation of pRb and attenuation of E1F2, thereby blocks transcription induced by E2F1. p21 also inhibits the activity of CDK2‐cyclin A and CDK1‐cyclin B which are necessary for orderly progression through S phase and G2/M, respectively. P53 induced by quercetin leads to induction of cell cycle inhibitors such as p21 and p27. p27 can influence cell cycle in several ways. In particular, p27 blocks the activity of CDK4‐cyclin D and CDK6‐cyclin D complexes, causing G1 cell cycle arrest.

Cell cycle progression is vital for cellular homeostasis and interaction between cyclins, cyclin‐dependant kinases (CDKs), and CDK inhibitors (CDKIs) is necessary to insure its orderly progression 39. Quercetin causes cell cycle arrest through its influence on several target proteins such as p53, p21, p27, cyclin B, cyclin D, and cyclin‐dependent kinases (Fig. 5). Quercetin preferably triggers cell cycle arrest at G2/M phase through induction of p73 and p21 and inhibition of cyclin B, both at the transcription and translation levels. Quercetin is also able to downregulate the cyclin B1 and cyclin‐dependent kinase‐1 (CDK‐1) which are required for orderly progression through G2/M phase of cell cycle 40. However, quercetin‐induced G1/S arrest is not less common and occurs through the induction of p21 and simultaneous phosphorylation of the retinoblastoma protein (pRb), which inhibits the G1/S cell cycle progression by blocking E2F1^41^. Depending upon the cell type quercetin can arrest cells even at G1 phase. For example, quercetin arrests HepG2 cells at G1 through induction of CDK inhibitors (p21, and p27) and tumor suppressor p53 40 (Fig. 5).

Quercetin is a potent inhibitor of topoisomerase II (TopoII), a cell cycle‐associated enzyme needed for DNA replication and chromosome segregation. Quercetin inhibits TopoII directly by stabilizing double‐stranded breaks (DSBs) in the ternary DNA‐TopoII‐flavonoid complex, the so‐called DNA cleavage complex. Quercetin‐3‐O‐glucoside a natural occurring form of quercetin causes cell cycle and subsequently apoptosis in hepatocellular carcinoma cells through its inhibitory influence on TopoII 42. However, quercetin‐induced TopoII inhibition causes mixed lineage leukemia gene (MLL) rearrangements that can lead to development of leukemia, raising concerns considering the probable genotoxic effects of quercetin on hematopoietic stem and progenitor cells (HSPCs) 43.

### Direct proapoptotic effects of quercetin

**Figure 6:**
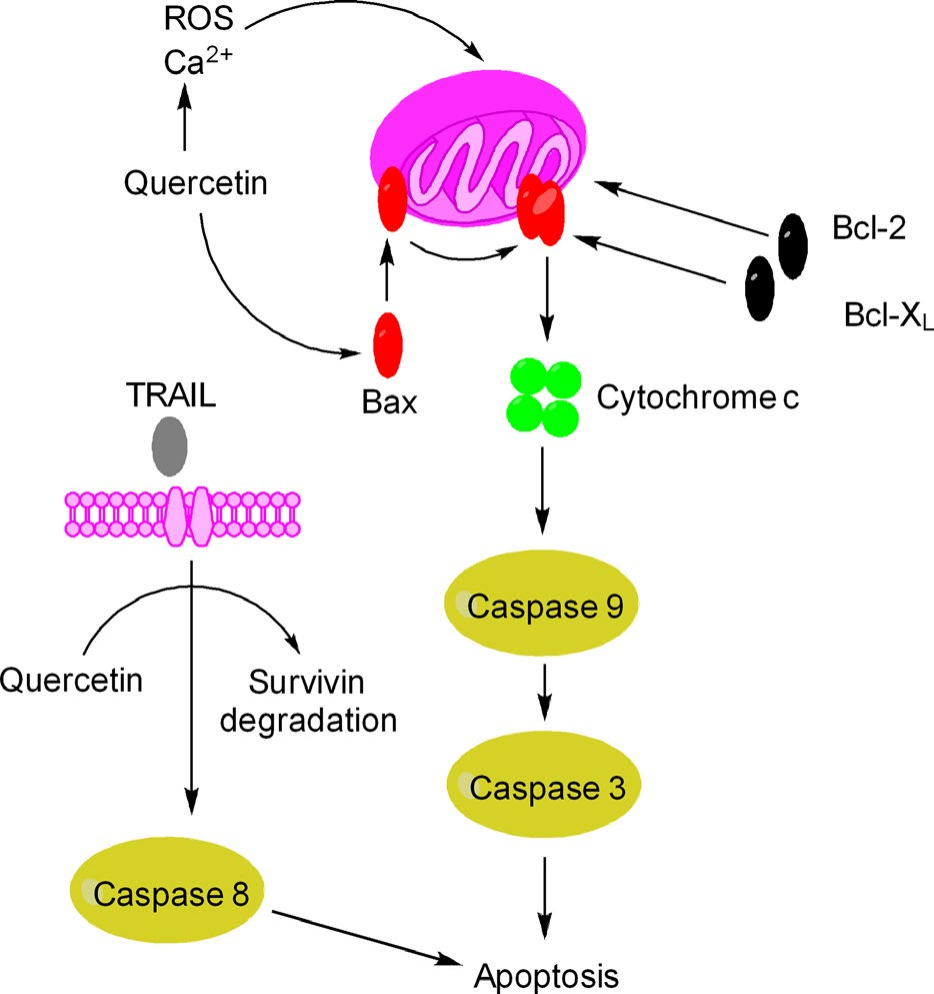
Molecular mechanism of quercetin‐induced apoptosis. Quercetin is a strong proapoptotic agent and activates both intrinsic and extrinsic pathways of apoptosis. Quercetin elevates the intracellular levels of ROS and Ca2+ leading to depolarization of mitochondrial membrane potential, release of cytochrome c and activation of caspases such as caspase 9 and caspase 3. Alternatively, quercetin promotes translocation of Bax from cytosol into mitochondria membrane leading to depolarization of mitochondrial membrane potential and subsequently induction of apoptosis. Penetration of Bax into mitochondrial membrane is normally inhibited by Bcl‐2 and Bcl‐XL proteins. Quercetin can promote TRAIL‐induced apoptosis by stimulating degradation of survivin and activation of caspase 8.

Apoptosis plays a central role in chemoprevention strategies. Quercetin can activate both intrinsic and extrinsic pathways of apoptosis. Quercetin activates intrinsic pathway of apoptosis (e.g., in MDA‐MB‐231 cells) through Ca^2+^‐mediated dissipation of mitochondrial membrane potential (MMP) and activation of caspase‐3, ‐8, and ‐9 44 (Fig. 6). Alternatively, quercetin can activate apoptosis (e.g., in HepG2 cells) through redistribution of Bcl‐2 family proteins, increased translocation of Bax to the mitochondrial membrane, activation of caspases, and concomitant blockade of the PI3K/Akt and ERK signals 18, 45 (Fig. 6). Quercetin‐induced apoptosis in HepG2 cells can be observed in animal models as well 46. Activation of intrinsic pathway of apoptosis by quercetin has also been reported in case of human epidermoid carcinoma KB and KBv200 cells and HT‐29 cells 47.

Quercetin influences extrinsic pathway of apoptosis (e.g., in HER2‐expressing breast cancer cells) through the activation of caspase‐8/caspase‐3 apoptotic cascade and inhibition of STAT3 signaling 48. Quercetin promotes TRAIL‐induced death‐receptor (DR4/DR5)‐mediated apoptosis in glioma cells through degradation of survivin 49 (Fig. 6). It is believed that quercetin enhances survivin degradation through increased proteasomal activity and repression of phosphorylated Akt. Both TRAIL and quercetin reveal minimal toxicity in normal, nontransformed cells. It has been recognized that normal, nontransformed cells continue to behave normally upon quercetin exposure. For example, human HaCaT keratinocytes and Hs68 human skin fibroblasts reveal no cytotoxicity at concentrations of which are otherwise cytotoxic to B16F10 and A375 melanoma cells 18. In another study, it has been revealed that quercetin inhibits breast cancer cells (e.g., T47D and EAC) without causing cell death in normal cells (293T and MEF1) 50.

### Quercetin and modulation of cell signaling networks

Quercetin has multiple intracellular targets and can elicit different response in different cell types due to inhibition/activation of multiple intracellular signaling networks 51.

#### Quercetin inhibits Wnt/β‐Catenin/T‐cell factor signaling

Wnt/*β*‐catenin/Tcf signaling plays a central role in early stages of cancer development, cellular growth processes, and apoptosis 52.Quercetin induces apoptosis in many cell types through inhibition of *β*‐catenin/Tcf signaling 53. Quercetin disrupts the association of *β*‐catenin with Tcf‐4 proteins and simultaneously retards the nuclear translocation of *β*‐catenin and Tcf‐4 proteins, causing decline in *β*‐catenin/Tcf transcriptional activity 53. Quercetin‐induced inhibition of *β*‐catenin nuclear translocation has been reported in triple negative breast cancer (TNBC), a highly lethal form of cancer that lacks estrogen, progesterone, and epidermal growth factor 2 receptors 54. Quercetin is a peculiar molecule, in that, it can inhibit epithelial‐to‐mesenchymal transition (EMT) but stimulates mesenchymal‐to‐epithelial transition (MET) in TNBC. For example, quercetin causes induction of E‐cadherin and suppression of vimentin levels. At the molecular level, quercetin inhibits nuclear accumulation of *β*‐catenin and inhibition of *β*‐catenin regulated genes such as cyclin D1 and c‐Myc, which in turn blocks metastatic behavior of triple negative breast cancer, which may have therapeutic significance in future 54. More recently, quercetin has been shown to suppress the metastatic behavior of lung cancer through the inhibition of Snail‐mediated EMT 55.

#### Quercetin targets p53 activity in favor of increased cell apoptosis

Quercetin plays a cardinal role in regulation of p53‐mediated apoptosis. Quercetin promotes p53 phosphorylation (e.g., in HepG2 cells) and stabilizes it both at transcription and translation levels, which in turn stimulates p21 and suppresses cyclin D1 in favor of increased cell apoptosis and cell cycle arrest 56 (Fig. 5). Alternatively, p53 contributes to quercetin‐induced NAG‐1 (nonsteroidal anti‐inflammatory drug‐activated gene‐1) expression, causing apoptosis (e.g., in HCT116 colon cancer cells) 57. In addition to p53, many other transcription factors (such as EGR‐1, Sp1, and PPAR*γ*) are involved in quercetin‐induced expression of NAG1 58.

**Figure 7:**
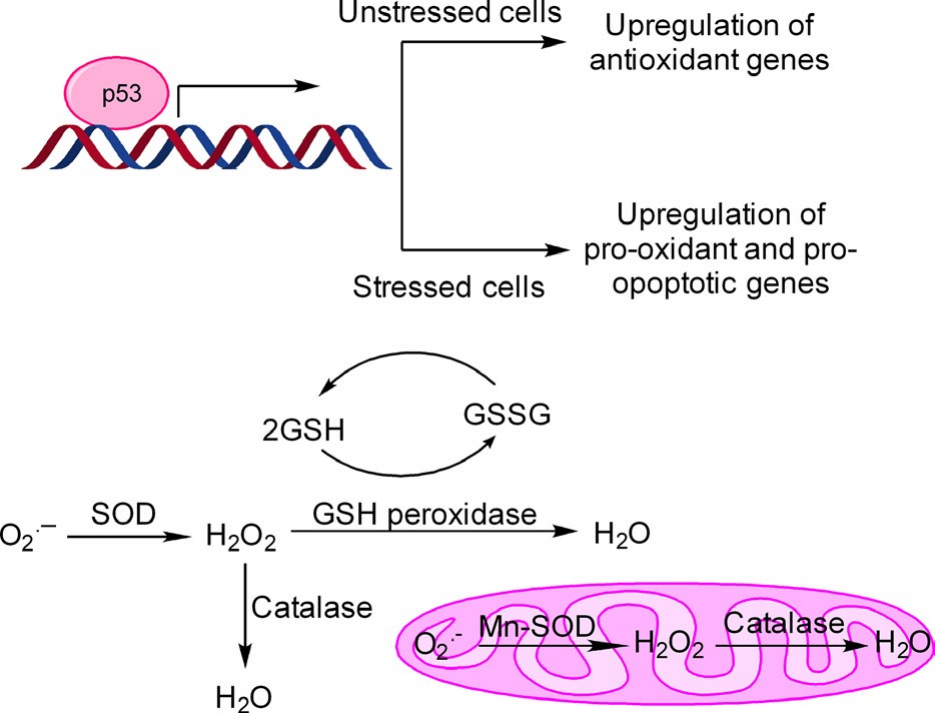
Effect of p53 on redox homeostasis and ROS production. p53 is a redox‐sensitive transcription factor and influences intracellular antioxidant system both in unstressed/ low‐stressed cells and stressed cells. In case of unstressed or low‐stressed cells, p53 upregulates antioxidant defense enzymes such as GPX1, Mn‐SOD, and catalase to maintain redox homeostasis. Each of these enzymes catalyzes a specific reaction to detoxify ROS. However, during high oxidative stress condition, p53 induces the transcription of many pro‐oxidant and proapoptotic genes (such as Bax, Puma, Noxa) with the consequent release of cytochrome c from mitochondria, activation of caspases and apoptosis.

It is interesting to know that when p53 is inhibited, cancer cells become vulnerable to quercetin‐induced apoptosis 59. A new model, based on the cardinal role of p53 in redox homeostasis, has been propounded to explain this observation 60 (Fig. 7). It is believed that end consequence of p53 activation in cell depends highly on the nature and intensity of redox stress. If there is no or minimal redox stress, p53 elevates enzymatic antioxidant defense system including GPX1, Mn‐SOD2, and catalase, which together detoxify ROS (Fig. 7). However, in case of high redox stress, p53 promotes transcription of many pro‐oxidant and proapoptotic genes such as Bax, Puma, and Noxa, leading to induction of apoptosis 61. More recently, it has been recognized that quercetin‐induced ROS formation and stabilization of p53 is involved in selective death of undifferentiated human pluripotent stem cells (hPSCs) raising the possibility that quercetin might be useful in reducing the chances of teratoma development during hPSC‐based cell therapy 62.

#### Inhibition of expression of Ras proteins

Ras is a small GTPase and a vital component of Ras‐Raf‐MEK‐ERK signaling 63. Thirty‐three percentage of human cancers harbors activation mutations in Ras isoforms (H‐Ras, K‐Ras, and N‐Ras). It has been noted that quercetin can selectively degrade oncogenic Ras in H‐Ras‐transformed colon cells without inducing degradation on wild‐type Ras 64. This specificity in quercetin‐induced degradation of K‐ and H‐Ras oncoproteins raises possibility that this natural molecule can be used for therapeutic targeting of Ras‐driven tumors. Quercetin‐induced preferential degradation of Ras oncoproteins is probably due to the activation of proteosomal machinery and induction of autophagy 64. Raf and MEK protein kinases, the downstream effectors of Ras, are also inhibited by quercetin 28.

#### Quercetin and PI3K inhibition

**Figure 8:**
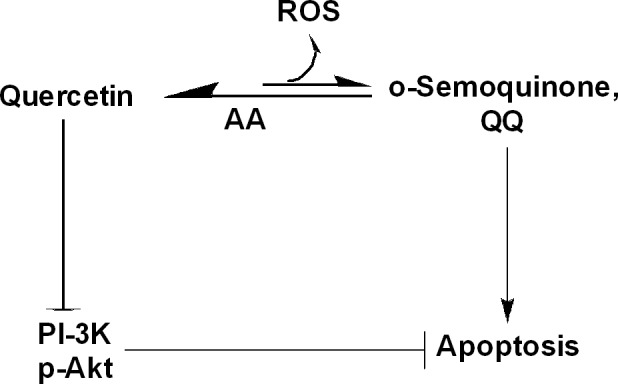
Influence of quercetin on PI3K/Akt pathways and ROS formation. Quercetin is a potent inhibitor of PI3K/Akt and an important trigger for formation of reactive intermediates, such as o‐semoquinone, quercetin–quinone products (QQ), and other forms ROS. Both PI3K inhibition and reactive intermediates formed from degradation of quercetin contribute to quercetin‐induced cell death. However, stabilization of quercetin with ascorbic acid (AA) paradoxically reduces apoptosis, but enhanced efficacy of quercetin‐induced PI3K inhibition.

Quercetin is a potent inhibitor of PI3K and strong inducer of reactive intermediates, such as o‐semiquinones, quercetin‐quinone products (QQ), and other ROS 65, 66 (Fig. 8). Therefore, the effect of quercetin on induction of apoptosis (e.g., in human HaCaT keratinocytes) is often due to combination of PI3K inhibition and generation of reactive intermediates. Thus, when quercetin degradation is retarded using ascorbic acid, PI3K inhibition is markedly enhanced; however, cell death is reduced paradoxically 66. Thus, the chemopreventive action of quercetin is decided both by the inhibition of PI3K and by its ability to form reactive intermediates. More recently, quercetin has also been show to exert chemopreventive effects in UVB‐irradiated B16F10 cells through the inhibition of PI3K‐Akt survival pathway 18.

#### Quercetin and inflammation

Chronic inflammation recruits immune sensitive cells (e.g., neutrophils, monocytes, and eosinophils) to the site of damaged tissue, creating a microenvironment with a collection of signaling molecules (e.g., arachidonic acid, cytokines, chemokines, and free radicals) which promote tumor invasion, metastasis, and angiogenesis 67. Chronic inflammation causes immunosuppression and can contribute to cancer development 68. Quercetin can inhibit cancer development through inhibition of inflammation‐producing enzymes (cyclooxygenase (COX), lipoxygenase (LOX), and PKC) and subsequent reduction in production of proinflammatory mediators 69. For example, quercetin suppresses early carcinogenesis in rat model of colon cancer through its inhibitory influence on iNOS, COX‐1, and COX‐2 expressions, all of which are upregulated in colon cancer 55.

#### Quercetin and NF‐κB/AP‐1 signaling

Nuclear factor *κ*B (NF‐*κ*B) and activator protein 1 (AP‐1) have been implicated cancer development and evasion of apoptosis 70. Both NF‐*κ*B and AP1 regulate expression of plethora of genes that are involved in cellular growth processes, adaptation, and differentiation. Quercetin is a potential inhibitor of NF‐*κ*B and AP1, which contributes to its growth inhibitory and/or chemopreventive effects 71. Quercetin inhibits growth of H460 lung cancer cells through NF‐*κ*B inhibition and induction of death receptors and cell cycle inhibitors 72. Quercetin inhibits transformations of c‐Fos overexpressing epithelial cells through inhibition of c‐Fos/AP1 complex 73.

#### Quercetin and endoplasmic reticulum (ER) stress

Cancer cells often develop ER stress due to altered protein homeostasis in the lumen of ER 74. To overcome this stress, cancer cells have evolved an adaptive mechanism known as the unfolded protein response (UPR) which plays a pivotal role in cancer development and chemoresistance 75. The UPR is primarily cytoprotective in nature, but under chronic ER stress conditions, apoptotic components of UPR are activated 76. The UPR is executed by three ER stress sensors‐ inositol‐requiring enzyme 1 (IRE1), activating transcription factor 6 (ATF6), and RNA‐dependent protein kinase (PKR)‐like ER kinase (PERK) and all these ER stress sensors are activated by quercetin in ovarian cancer 77. Quercetin‐induced ER stress in ovarian cancer is not well understood. It is believed that quercetin induces ER stress‐mediated apoptosis in ovarian cancer cells via p‐STAT/Bcl‐2 signaling axis 78. More recently, quercetin has been reported to impair Hsp70/IRE‐1*α* signaling axis and downregulate the expression of Hsp70 in U937 monoblastic leukemia cells 79. Hsp70 protects ER stressed cells from apoptosis. When impaired, Hsp70‐IRE1*α* axis turns a mild ER stress into a severe ER stress and induces the expression of CHOP, a bZIP transcription factor implicated in ER stress‐mediated apoptosis.

#### Quercetin and autophagy

Autophagy, a highly conserved catabolic process, usually imparts survival advantage to cancer cells in response to cell intrinsic and extrinsic stressors. However, if excessively induced, it can trigger nonapoptotic type of cell death called autophagy‐induced cell death (type II PCD) 80. Quercetin is a potent inducer of autophagy in many cancer cell lines 81, 82, 83 and can induce autophagy‐mediated apoptosis in certain cancer cells through the inhibition of proteasomal activity and mTOR signaling 84. mTOR inhibition activates autophagy signaling 85. Quercetin‐induced proteosome inhibition and blockade of mTOR/4EBP1/p70S6 kinase activity play a vital role in induction of cell death 86. However, in case of some gastric cancer cells (e.g., AGS and MNK28), quercetin induces protective autophagy. In these cells, administration of chloroquine (the autophagy inhibitor) or targeted deletion of Atg5 or beclin‐1 using specific siRNA promoted cell death, indicating that autophagy protects these cells against the quercetin‐induced apoptosis 87. At the molecular level, quercetin‐induced apoptosis can be modulated by Akt‐mTOR signaling and hypoxia‐induced factor 1*α* (HIF‐1*α*) signaling. Quercetin inhibits cell proliferation and induces autophagy in U87 and U251 glioma cells 88. Therefore, quercetin should be administered for chemoprevention of malignant glioma at late stage. In addition, quercetin promotes TRAIL‐induced apoptosis in human lung cancer cells via autophagy flux activation, degradation of p62, and increased LC3B conversion, thereby raising the possibility of using quercetin in combination with TRAIL as combination chemopreventive strategy for lung cancer 89.

## Quercetin and Induction of Phase II Metabolizing Enzymes

The cancer chemopreventive effects of quercetin are, atleast in part, because of its functional role in induction of phase II drug metabolizing enzymes 90, 91. Phase II drug‐metabolising enzymes are mainly transferases and include glutathione S‐transferase *α* 2 (GSTA‐2), NAD(P)H:quinone oxidoreductase (NQO‐1), *γ*‐glutamate cysteine ligase (*γ* ‐GCLC and *γ* ‐GCLM), and heme oxygenase‐1 (HO‐1).

## Quercetin and Ultraviolet B UV(B)‐induced Signaling

**Figure 9:**
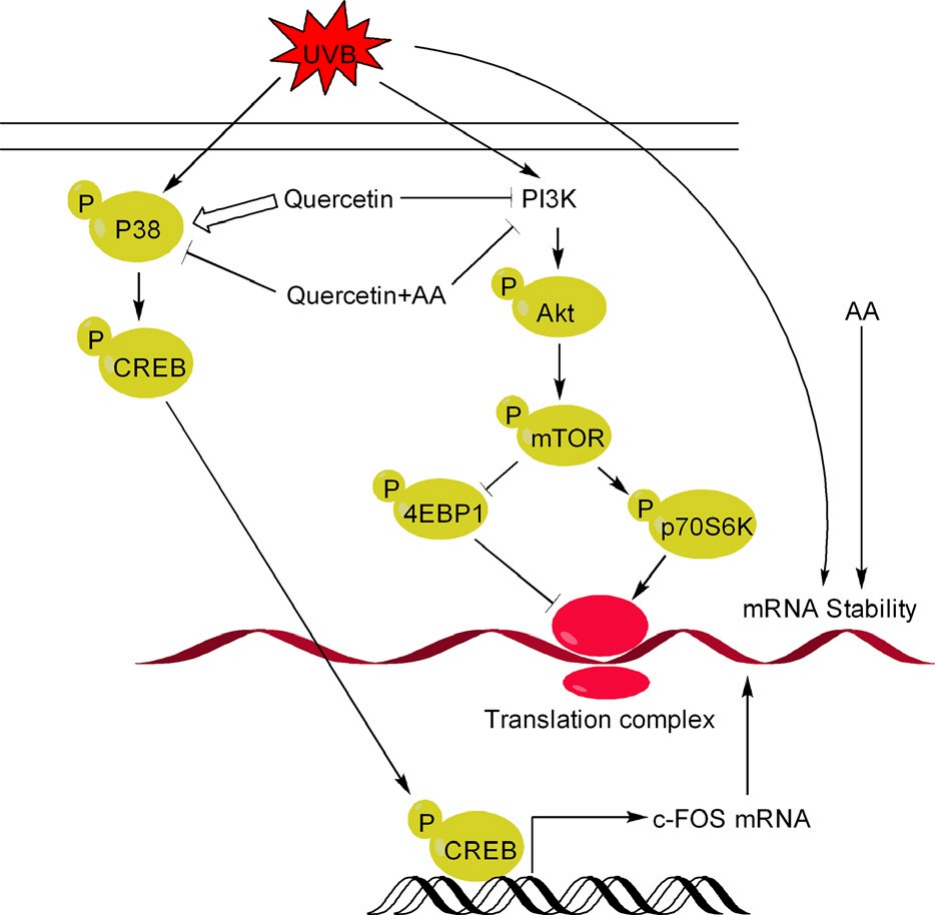
Influence of quercetin on c‐Fos mRNA level and c‐Fos expression in response to UVB exposure.

Ultraviolet (UV) is an important etiologic factor of skin cancer development 92. Quercetin plays a pivotal role in triggering cell death in UV(B)‐irradiated human HaCaT keratinocytes 66. HaCaT are p53‐mutated premalignant cells that are widely used to understand the chemopreventive effects of natural agents in response to UV (B) irradiation. Quercetin, apart from inhibiting the growth of UVB‐irradiated HaCaT cells, can inhibit the growth of B16F10 and A375 melanoma cells as well 18. It is interesting to note that while quercetin is advantageous as a chemopreventive agent, it can contribute to cancer development through induction expression c‐Fos expression in response to UV B exposure 72. Quercetin‐induced increase in c‐Fos expression is due to activation of p38 and CREB in response to UVB irradiation (Fig. 9). Notably, ascorbic acid (AA) blocks p38 and CREB activation but promotes c‐fos mRNA stability, with the result c‐fos mRNA level increases in cells receiving combination treatment of quercetin and ascorbic acid. However, combination treatment of quercetin and ascorbic inhibits PI3K and its downstream effector mTOR with better efficacy than quercetin, resulting in inhibition of c‐fos translation even in the presence of increased mRNA levels. Ascorbic acid prevents degradation of quercetin. Therefore, quercetin alone inhibits this pathway less effectively resulting in increased c‐Fos translation due to availability of high levels of c‐Fos mRNA. Quercetin must be stabilized to improve its photochemopreventive efficiency.

## Chemopreventive Effects of Quercetin in Animal Models

The role of quercetin in cancer chemoprevention has been investigated in benzo(a)pyrene‐induced mouse model of lung cancer. It was observed that administration of quercetin before initiation of cancer prevented the subsequent carcinogen‐induced lung cancer as indicted by an increment in activity of antioxidant enzymes (superoxide dismutase, catalase, GSH peroxidase, GSH‐S‐transferase, GSH reductase) and a decline in lipid peroxidation 93.

Similarly, quercetin can reverse the formation of N‐nitrosodimethylamine (DEN)‐induced rat liver preneoplastic lesions and protect these animals against the development of hepatocellular carcinoma 94.

## Conclusion

It is now clear that quercetin is a potent chemopreventive molecule with multifaceted effects and intracellular targets. Quercetin is an ideal therapeutic tool for chemoprevention of cancer and can specifically activate apoptosis in cancer cells, without inhibiting cellular growth of normal cells. However, quercetin has relatively low bioavailability and poor aqueous solubility that hamper its use as a therapeutic agent. The bioavailability of quercetin can be significantly enhanced if it is consumed as a component of whole food. Till date, there are limited epidemiological studies that support its therapeutic efficacy. The preclinical data of quercetin is promising and can be greatly enhanced by the identification, development, and authentication of early and reliable biomarkers for cancer development. Quercetin is capable of inducing different responses that need to be characterized to understand how this natural molecule might be useful in cancer chemoprevention.

## Conflict of Interest

The authors have declared that there are no conflicts of interests.
